# Ras-association domain family 1C protein promotes breast cancer cell migration and attenuates apoptosis

**DOI:** 10.1186/1471-2407-10-562

**Published:** 2010-10-18

**Authors:** Mark E Reeves, Scott W Baldwin, Melissa L Baldwin, Shin-Tai Chen, Jeremy M Moretz, Robert J Aragon, Xinmin Li, Donna D Strong, Subburaman Mohan, Yousef G Amaar

**Affiliations:** 1 Surgical Oncology Laboratory, 11201 Benton Street (151), Loma Linda VA Medical Center; California, 92350, USA; 2 Musculoskeletal Disease Center, 11201 Benton Street (151), Loma Linda VA Medical Center; California, 92350, USA; 3 Department of Surgery, Loma Linda University, 11175 Campus St, Loma Linda, California, 92350, USA; 4 Department of Pathology, University of California, 1000 Veteran Avenue, Los Angeles, CA 90095, USA

## Abstract

**Background:**

The Ras association domain family 1 (RASSF1) gene is a Ras effector encoding two major mRNA forms, RASSF1A and RASSF1C, derived by alternative promoter selection and alternative mRNA splicing. RASSF1A is a tumor suppressor gene. However, very little is known about the function of RASSF1C both in normal and transformed cells.

**Methods:**

Gene silencing and over-expression techniques were used to modulate RASSF1C expression in human breast cancer cells. Affymetrix-microarray analysis was performed using T47D cells over-expressing RASSF1C to identify RASSF1C target genes. RT-PCR and western blot techniques were used to validate target gene expression. Cell invasion and apoptosis assays were also performed.

**Results:**

In this article, we report the effects of altering RASSF1C expression in human breast cancer cells. We found that silencing RASSF1C mRNA in breast cancer cell lines (MDA-MB231 and T47D) caused a small but significant decrease in cell proliferation. Conversely, inducible over-expression of RASSF1C in breast cancer cells (MDA-MB231 and T47D) resulted in a small increase in cell proliferation. We also report on the identification of novel RASSF1C target genes. RASSF1C down-regulates several pro-apoptotic and tumor suppressor genes and up-regulates several growth promoting genes in breast cancer cells. We further show that down-regulation of caspase 3 via overexpression of RASSF1C reduces breast cancer cells' sensitivity to the apoptosis inducing agent, etoposide. Furthermore, we found that RASSF1C over-expression enhances T47D cell invasion/migration *in vitro*.

**Conclusion:**

Together, our findings suggest that RASSF1C, unlike RASSF1A, is not a tumor suppressor, but instead may play a role in stimulating metastasis and survival in breast cancer cells.

## Background

The Ras association domain family 1 (RASSF1) proteins are postulated to function as Ras effectors and to affect cell growth. The RASSF1 gene resides on chromosome 3p21.3, a region that often undergoes homozygous or heterozygous deletions and hypermethylation-induced suppression in many human cancers [1,2]. The RASSF1 gene encodes multiple isoforms derived by alternative promoter selection and alternative mRNA splicing [1,2], with two major isoforms called RASSF1A and RASSF1C. The RASSFIA protein (340 amino acids) contains an amino-terminal diacyl glycerol binding domain (C1 domain), an ataxia telangiectasia mutated (ATM) phosphorylation site, and a carboxy-terminal putative Ras association (RA) domain. The RASSFIC protein (270 amino acids) contains the ATM phosphorylation site and the RA domain, but not the C1 domain [1,2].

RASSF1A is a tumor suppressor gene which is epigenetically inactivated by cytidine methylation in many human solid tumors. It has been reported that in 80 to 100% of lung cancer cell lines and tumors [1-4], 49 to 62% of breast cancers [3,5], 67 to 70% of nasopharyngeal cancers [6], 90% of hepatocellular carcinomas [7], 91% of renal cell carcinomas [8], and 70% of prostate cancers [9,10], the RASSF1A gene, but not the RASSF1C gene, is inactivated. In addition, RASSF1A over-expression reduces colony formation, suppresses anchorage independent growth, inhibits tumor growth in nude mice, and inhibits cell growth by inducing G1-S phase cell cycle arrest and by blocking cyclin D accumulation [2,8,11,12]. Studies of RASSF1A knockout mice showed that RASSF1A -/- and RASSF1A+/- mice exhibit enhanced tumor multiplicity and tumor size compared to wild type animals upon exposure to the chemical carcinogens benzo(a) pyrene and urethane [13].

The RASSF1C isoform differs from the RASSF1A isoform by having a distinct N-terminus and lacking the diacyl glycerol binding domain. Unlike RASSF1A, RASSF1C has not been extensively studied, and very little is known about its role in cell growth, survival, and metastasis. In contrast to RASSF1A, RASSF1C is expressed in almost all human solid tumors. The majority of published literature indicates that RASSF1C has no tumor-suppressor activity [2,9,11,12,14]. However, some reports suggest that RASSF1C may function as a tumor suppressor in ovarian, prostate, renal cancer cells [15-17]. We have recently identified RASSF1C as an Insulin-like Growth Factor Binding Protein-5 (IGFBP-5) interacting protein and have shown that silencing of RASSF1C expression resulted in a significant decrease in osteosarcoma and lung cancer cell proliferation [18,19]. We have also shown that over-expression of RASSF1C increased cell proliferation of the lung cancer cell line NCI H1299, suggesting a growth promoting role for RASSF1C in lung cancer cells [19]. In this paper we report on the effects of silencing and over-expressing RASSF1C on human breast cancer cell growth, apoptosis, and invasion, and on the identification of novel RASSF1C target genes.

## Methods

### Cell culture

The human breast cancer cell lines Hs578T, MDA-MB231 and T47D were obtained from American Type Culture Collection ATCC, Manassas, VA). Cell culture was carried out as recommended by ATCC. Hs578T and MDA-MB231 cells were grown in DMEM supplemented with 10% calf bovine serum. T47D cells were grown in RPMI-1640 medium supplemented with 10% calf bovine serum and 0.2 units/mL insulin. The human mammary epithelial cell line AG1132B was obtained from Coriell Institute for Medical Research (Camden, NJ). Cell culture was carried out as recommended by the supplier.

### Transfection of cell lines with plasmid DNA

The MDA-MB231 and T47D cell lines were transfected with siRNA-RASSF1C and control plasmids as previously described [20]. Since the shRNA plasmids used in this study would target both RASSF1A and RASSF1C mRNAs, we utilized breast cancer cells that express RASSF1C but not RASSF1A. Cells were plated at 20,000 and 50,000 cells per well in the appropriate medium with 10% calf serum in 24 and 6 well culture dishes, respectively. After 24 hr, the cells were transfected with 1 μg/ml plasmid DNA using Lipofectamine (Invitrogen, Carlsbad, CA) using recommended conditions. 48 hr post-transfection, cells were collected and were used for RNA extraction. 200 ng of total RNA was used to prepare cDNA with the Omniscript kit (Qiagen, Valencia, CA). 100 ng of the reverse transcriptase reaction was used for PCR using the HotStart master mix (Qiagen, Valencia, CA) and PCR reactions were run with the following conditions: 95°C 1 min, 60°C for 30 sec, and 72°C for 30 sec for 35 cycles.

### Alamar blue assay

Cells were treated with either siRNA-RASSF1C (suppresses RASSF1C expression) or control plasmid for 48 hr, and cell proliferation was measured by the alamar blue assay as previously described [18,19].

### 3H-Thymidine incorporation

MDA-MB231, T47D, and AG1132B cells were treated with either siRNA-RASSF1C (suppresses RASSF1C expression) or control plasmid for 18 hr. 3H-thymidine was then added and cells were labeled for 6 hr before cultures were terminated and 3H-thymidine incorporation was assayed as previously described [19].

### Construction of a tet-inducible expression system that expresses RASSF1C

In order to over-express RASSF1C cDNA in human breast cancer cells in a regulated fashion, we chose to use a doxycycline (dox)-inducible Murine Leukemia Virus based retroviral vector that was developed in house (Chen et. al., manuscript in preparation). Using the YFP-RASSF1C plasmid [18,19] as a template, the full-length HA-RASSF1C coding sequence was cloned using the forward primer: 5' GGG TCG ACG CGG CCG CC ATGTACCCATACGATGTTCCGGATTACGCT GGC GAG GCTGAAACACCTT 3' and the reverse primer: 5' CGG GAT CC TCA CCC AAG GGG GCA GGC GTG CAG 3', and the HA-IGFBP-5 coding sequence was cloned using the forward primer: 5' GGG TCG ACG CGG CCG CC ATGTACCCATACGATGTTCCGGATTACGCT GGC TCC TTC GTG CAC TGC GA 3', and the reverse primer: 5' CGG GAT CC TCA CTC AAC GTT GCT GCT GTC GAA 3' flanked by NotI and BamHI restriction enzyme sites, respectively. The NotI and BamHI sites in the pGYT plasmid were used to insert the RASSF1C cDNA sequence down stream of the TetO and mammalian promoter. The correct cDNA sequence and orientation were confirmed by sequencing the pGYT-HA-RASSF1C plasmid. This plasmid then was used to produce VSV-G pseudotyped MLV-based vector as described [20].

MDA-MB231 and T47D breast cancer cell lines were seeded at 1 × 10^5 ^cells/well in 6-well plates. After 24 hr of incubation, the cells were transduced with MLV-based vectors rtTA-GYT (vector without transgene), rtTA-GYT-GFP, and rtTA-GYT-HA-RASSF1C with different MOI in 6-well plates, using 2 or 3 serial infection cycles as described [20]. After 1-4 days, cells were treated with up to 1 × 10^-6 ^M doxycycline (dox) for 48 hr. HA-RASSF1C expression was assessed by Western blot analysis using anti-HA antibody. We tested the MLV-GFP vector expression in human breast cancer cell lines and demonstrated that a 10 fold induction of GFP expression can be achieved with a dox concentration of 1 ug/ml.

### RNA isolation and RT-PCR analysis

Total RNA from human cell lines was isolated from confluent cultures using the Absolutely RNA Microprep Kit (Stratagene, La Jolla, CA). 1 μg of total RNA was used to set up reverse transcriptase (RT) reactions using the superscript kit (Qiagen, Valencia, CA) and the RT reactions were subsequently used to set up real-time PCR reactions using 1 μl of RT as a template. 1 μg of total RNA was used to perform reverse transcription reactions (RTs) and 1 ul of the RT reaction was used to set up qRT-PCR reactions in triplicates using RASSF1A and RASSF1C specific primer. RASSF1A forward primer is 5'GGCGTCGTGCGCAAAGGCC and RASFF1C forward primer: is 5'CTGCAGCCAAGAGGACTCGG 3', and the reverse primer for both RASSF1A and 1C is 5'GGGTGGCTTCTTGCTGGAGGG 3' [2]. The real-time PCR reactions were set up using SYBR green PCR master mix (Bio-Rad, Hercules, CA) and the PCR reactions were run using the Opticon 2 PCR machine (Bio-Rad). The PCR reactions were run using the following protocol: 1. incubate at 95°C for 10 min, 2. incubate at 95°C for 15 sec, 3. incubate at 60°C for 30 sec, 4. incubate at 72°C for 30 sec, 5. go to step 2 for 39 more cycles, 6. melting curve from 60°C to 95°C, read every 1.0°C.

### Western blot analysis

Western blot analysis was carried out using the Odyssey^® ^Infrared System (LI-COR Biosciences, Lincoln, NE). Anti-caspase 3, P-ERK1/2, total ERK1/2, and -GHR antibodies were purchased from Santa Cruz Biotechnology (1: 250-1000, Santa Cruz, CA), the anti-HA antibody was purchased from Covance (1:1000; Berkeley, CA), the anti-CXCR4 antibody was purchase from Millipore (1:500; Temecula, CA), and the anti-trans glutaminase 2 (1:1000; TGM2) antibody was purchased from Sigma (Saint Luis, MO). Fluorescently labeled secondary antibodies were purchased from LI-COR Biosciences (1:5000). Cells were collected and lysed in RIPA buffer supplemented with protease inhibitors. 30-40 ug of protein was separated on 12% SDS-PAGE gels and transferred to nitrocellulose membranes. The membranes were blocked overnight at 4°C in TBST and dried milk. Incubation with antibodies was carried out in Odyssey^® ^Infrared System blocking buffer (diluted 1:10 in PBST blocking buffer).

### Microarray analysis

Hybridization of 12 μg of labeled cRNA to an Affymetrix U133 plus 2.0 chip was carried out in triplicates and data analyses were carried out at the UCLA Microarray facility core, Department of Pathology. The control sample is RNA from T47D cells stably transduced with MLV-backbone (T47D-BB) and the experimental sample is RNA from T47D cells stably transduced with MLV-RASSF1C (T47D-1C). Prior to RNA isolation, T47D-BB and T47D-1C cells were treated with 1 μg/ml doxycycline for 48 hr. Data analysis was performed using dChip [21]. Thresholds for selecting significant genes were set at a relative difference > = 1.5-fold (or/and 2-fold), absolute signal difference > = 50, and p < 0.05. Genes that met all three criteria were considered as significant changes. Comparison results with False Discovery Rate (FDR) <5% was considered as a valid analysis. The microarray data has been deposited in the Gene Expression Omnibus (GEO) data base and the accession number is GSE24473.

### Primers used to validate selected RASSF1C target genes

Caspase 3 gene primers were purchased from realtimeprimers.com (Elkins Park, PA). Other gene primers were as follows: Cyclophilin forward primer 5' GCATACAGGTCCTGGCATCT3 and reverse 5'TCTTGCTGGTCTTGCCATTC3'; CREG forward primer: 5'GTGCCCTATTTCTACCTGAGCC 3' and reverse primer 5'AGCATTATGTGAACACAAAGGGG 3'; CXCR4 forward primer: ATGAAGGAACCCTGTTTCCGT and reverse primer 5' AGATGATGGAGTAGATGGTGGG 3'; EGR1 forward primer: 5'ATGAAGGAACCCTGTTTCCGT 3'and reverse primer 5' ATGATGGAGTAGATGGTGGG 3'; GHR forward primer 5' GCTGCTGTTGACCTTGGC 3' and reverse primer 5'ACCTCATCTGTCCAGTGG 3'; MRAS forward primer: 5'TTTGTGCCTGACTATGACCCC 3' and reverse primer 5' TGACGGAGTAGACGATGAGGA 3'; and HOXA1 forward primer: 5' CGCCTCAATACATTCACCACT 3', and reverse primer 5' CCAGCAGGACTGACCTGTTTT 3'. The RT-PCR reactions were carried out in triplicate and the fold change was calculated using the 2^-ΔΔC^_T _method [22].

### Infection of breast cancer cells with Mission^® ^lentiviral shRNA tranduction particles

Breast cancer cells were plated at 5000/well in 96-well plates 24 hrs before infection.

Cells were incubated with 8 μg/ml hexadimethrine bromide for two hours before virus particles were added. Cells were infected with Mission^® ^non-target shRNA control transduction particles or with multiple Mission^® ^lentiviral shRNA transduction particles (NMID: NM_007182) for silencing RASSF1C (Sigma, St. Louis, MO). Since the lentiviral shRNA Transduction Particles (NMID: NM_007182) used in this study would target both RASSF1A and RASSF1C, we used breast cancer cells that express RASSF1C but not RASSF1A. The infections were carried out using an MOI of at as outline in the supplier manual. Infected cells were selected in media containing 2 μg/ml puromycin for 2-4 weeks and then cells were harvested. Knockdown validation of RASSF1C expression was assessed by qRT-PCR using RASSF1C specific primers.

### Caspase 3 activity assay

Caspase 3 activity was assayed using the Apo3/7 caspase activity assay (Promega, Madison, WI). Cells were plated in 96-well plates at 5000 cells/well and the next day cells were treated with doxycycline, DMSO, etoposide at 45 umol/ml, or doxycycline and etoposide for 48 hr before cells were assayed for caspase 3 activity. Etoposide was purchased from Sigma and diluted in DMSO (Sigma) to a concentration of 45 mM and doxycycline was purchased from Invitrogen (Carlsbad, CA).

### DNA fragmentation assay

Breast cancer cells stably over-expressing RASSF1C were incubated for 14 days in presence of 1 μg/ml doxycycline before cells were used to isolate genomic DNA for DNA fragmentation analysis using an Apoptotic DNA Ladder Kit (Roche Applied Science, Mannheim, Germany). Apoptotic DNA ladder corresponding to genomic DNA isolated from lyophilized apoptotic U937 cells that were treated with 4 ug/ml camptothecin for 3 hrs that were provided with the kit used as a positive control for apoptosis.

### *In vitro *cell invasion assay

The 24-well plate BD BioCoat™ Matrigel™ Invasion Chamber (BD Biosciences. Bedfor, MA) was used to co-culture T47D breast cancer cells with human stromal cells, Hs27a according to the user manual. The Hs27a cells were seeded at 25,000 cells per well in the 24-well BD Falcon™ TC Companian Plate in DMEM supplemented with 10% calf bovine serum. T47D cells were seeded at a density of 25,000 cells in the Matrigel chambers, T47D-BB and T47D-1C were grown in RPMI-1640 medium supplemented with 10% calf bovine serum and 0.2 units/mL insulin. Then the chambers containing the T47D-BB and T47D-1C were transferred to the well containing the Hs27a stromal cells and incubated for 22 hours. MDA-MB231 and T47D cells were also seeded at a density of 25,000 cells in the Matrigel chambers and the chambers were transferred to wells containing either 40 ng/ml SDF-1 conditioned medium or control medium lacking SDF-1 and incubated for 22 hours. The cells in the lower surface of the membrane were fixed methanol and stained with 1% Toluidine blue per the user manual instructions. The stained membranes were photographed through the microscope and invading cells were counted.

### Statistics

Data are presented as mean values ± SEM and analyzed with Student's *t*-test. Values ≤0.05 were considered significant.

## Results

### Silencing of RASSF1C decreases breast cancer cell proliferation

**Figure 1:**
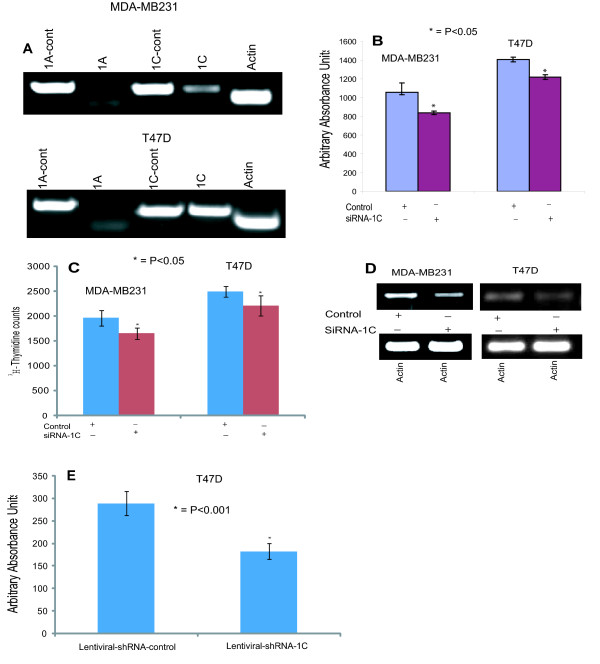
**(A) RT-PCR analysis of RASSF1A and RASSF1C expression in breast cancer cell lines (MDA-MB231 and T47D) using RASSF1A and RASSF1C specific primers. The RT-PCR results clearly show that RASSF1C mRNA is expressed in both cell lines**. Unlike RASSF1C, RASSF1A mRNA expression was not readily detected in MDA-MB23 and T47D cells. RASSF1A and RASSF1C cDNAs were used as templates as control for RASSF1A (1A-cont) and RASSF1C (1C-cont) primers and actin was used as an internal control. (B) MDA-MB231 and T47D breast cancer cells were transfected with psilencer-RASSF1C-258 (siRNA-1C) to silence RASSF1C and pSilencer (control) plasmids as previously described [18,19]. The proliferation of cells treated with siRNA-1C was significantly reduced (P < 0.05, t-test) compared to cells transfected with the empty pSilencer plasmid. (C) Transfected MDA-MB231 and T47D cells were also incubated with 3H-thymidine and 3H-thymidine incorporation was assayed as previously described [19]. Proliferation of cells treated with siRNA-1C was significantly reduced (P < 0.05 vs vector control, t-test) compared to cells transfected with control plasmid. Values are mean ? SEM of 8 replicas; experiments were performed at least three independent times. (D) RT-PCR analysis of RASSF1C expression in transfected MDA-MB231 and T47D either transfected with psilencer-RASSF1C-258 (siRNA-1C) or pSilencer plasmid alone (control). The RT-PCR results clearly show that RASSF1C mRNA was significantly reduced in cells treated with siRNA-1C compared to control. The amplification of actin as an internal control is the same in both treated and control cells. (E) T47D breast cancer cells were infected with Mission^® ^lentiviral-shRNA-control particles (lentiviral-control) and Mission^® ^lentiviral-shRNA-RASSF1C (lentiviral-shRNA-1C) to silence RASSF1C. The proliferation of cells infected with lentiviral-shRNA-1C was significantly reduced (P < 0.001, t-test) compared to cells infected with lentiviral-shRNA-control.

Because RASSF1C and RASSF1A are structurally similar, but appear to have opposing effects, it is possible that they may interact and modulate each other's effects. Therefore, prior to silencing RASSF1C mRNA, the endogenous RASSF1A and RASSF1C mRNA levels were measured in MDA-MB231 and T47D breast cancer cells. RASSF1C is readily detectable, while RASSF1A is barely detectable in both cell lines (Figure 1A).

Next, expression of RASSF1C was silenced with small interfering RNA (siRNA) technology. The siRNA-RASSF1C plasmid used in this study is one of three RASSF1C siRNA plasmids [18] that we previously demonstrated to consistently reduce HA-RASSF1C protein expression compared to non-target siRNA oligos as judged by Western blot analysis using anti-HA antibody [19].

Cells transfected with siRNA-RASSF1C plasmid showed a significant decrease (p < 0.05) in cell proliferation compared to cells transfected with control plasmid as judged by the alamar blue and the ^3^H-thymidine incorporation assays (Figure 1B and 1C). To confirm that the inhibitory effect of RASSF1C siRNA on cell number correlated with reduction of RASSF1C mRNA, RASSF1C mRNA levels were measured in MDA-MB231 and T47D cultures treated with siRNA-RASSF1C or control plasmid. Figure 1D shows that transient transfection with siRNA-RASSF1C reduced RASSF1C mRNA levels in these breast cancer cells. We have also confirmed our plasmid silencing data using Mission^® ^lentiviral shRNA transduction particles to silence RASSF1C expression in T47D cells (Figure 1E). These findings suggest that RASSF1C appears to be important in promoting breast cancer cell growth.

### Over-expression of RASSF1C in breast cancer cells does not inhibit breast cancer cell growth

**Figure 2:**
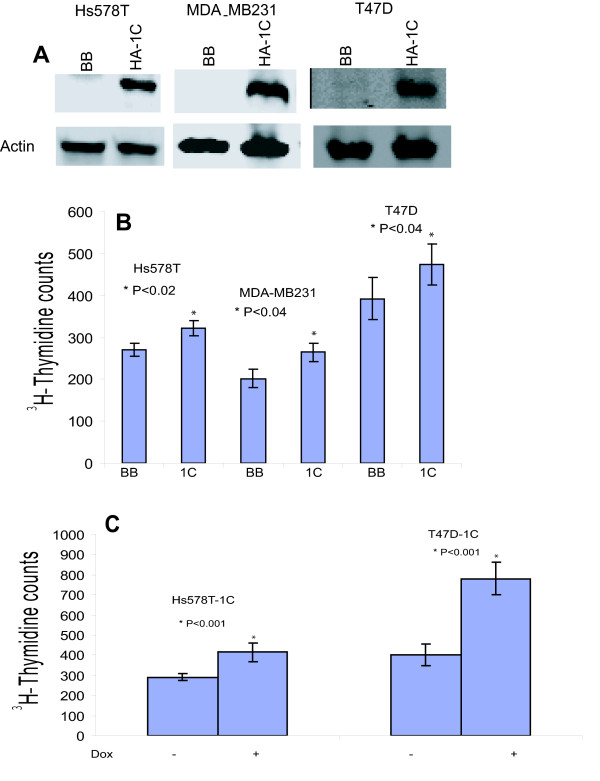
**(A) Western blot analysis of Hs578T, MDA-MB231 and T47D cells stably transduced with either empty MLV back bone (BB) or MLV-HA-RASSF1C (1C) and treated with 1 μg/ml doxycycline for 48 hr**. The anti-HA tag antibody detected a HA-RASSF1C fusion protein in cells transduced with 1C but not BB as expected. (B) Hs578T, MDA-MB231, and T47D cells stably transduced with an empty MLV-backbone (BB) or MLV-HA-RASSF1C (1C) vectors were treated with 1 μg/ml doxycycline for 48 hr. Cells were incubated with ^3^H-thymidine for 6 hr and ^3^H-thymidine incorporation was assayed as previously described [19]. (C) Hs578T-1C and T47D-1C treated with doxycycline showed an increase in cell proliferation compared to cells not treated with doxycycline, respectively. RASSF1C over-expression did not inhibit but rather caused a small and statistically significant increase in cell proliferation compared to cells stably transduced with empty MLV-backbone vector. Values are mean ± SEM of 8 replicates; experiments were performed at least three independent times.

To further elucidate the function of RASSF1C and demonstrate that RASSF1C is not a tumor suppressor, we carried out RASSF1C over-expression studies in breast cancer cells using a tet-inducible Mouse Leukemia Virus-based retroviral vector to express HA-tagged RASSF1C fusion protein. Cells were stably transduced with MLV-backbone (BB) or MLV-RASSF1C (1C) as outlined in Materials and Methods. Western blot analysis using an anti-HA-tag antibody to detect the HA-RASSF1C fusion protein verified that RASSF1C was over-expressed in cells transduced with the MLV-RASSF1C vector following treatment with 1 μg/ml doxycycline for 48 hr (Figure 2A). Over-expression of RASSF1C did not inhibit cell proliferation. Instead, it consistently resulted in a small but reproducibly and statistically significant increase in cell proliferation of Hs578T, MDA-MB231, and T47D cells stably transduced with MLV-HA-RASSF1C (1C) compared to an empty MLV-backbone (BB) as demonstrated by ^3^H-thymidine cell proliferation assays (Figure 2B and 2C). These findings demonstrate that RASSF1 C over-expression does not inhibit breast cancer cell growth and may suggest a potential role of RASSF1C in promoting cancer cell growth and progression.

### Assessment of RASSF1C expression in primary mammary epithelial cells and breast cancer cell lines

We assessed the expression of RASSF1A and RASSF1C in primary mammary epithelial (AG1132B) cells and established breast cancer cell lines using quantitative real time PCR (qRT-PCR). The RT-PCR reactions were carried out in triplicates and the fold change was calculated using the 2^-ΔΔC^_T _method [22]. Interestingly, RASSF1C expression was at least 6 fold higher and RASSF1A was at least 2.5 fold lower in the breast cancer cell lines (Hs578T, MDA-MB231, and T47D) compared to the normal mammary epithelial cells, AG1132B) (Table 1). The elevated expression of RASSF1C detected in established breast cancer cell lines compared to primary cells is indeed consistent with our hypothesis that RASSF1C, unlike RASSF1A, is a potential growth and survival factor in breast cancer.

**Table 1:** RASSF1A and RASSF1C mRNA expression in epithelial and breast cancer cell lines

Cell Line	Gene	Relative expression levels
AG1132B	RASSF1A	1
	RASSF1C	1
Hs578T	RASSF1A	-3.3
	RASSF1C	4
MDA-MB231	RASSF1A	-2.2
	RASSF1C	7
T47D	RASSF1A	-8
	RASSF1C	6

QRT-PCR analysis of total RNA from human primary mammary epithelial cells (AG1132B) and from established breast cancer cell lines, Hs578T. MDA-MB231, and T47D cells. 1 μg of total RNA was used to perform reverse transcription reactions (RTs) and 1 ul of the RT reaction was used to set up qRT-PCR reactions in triplicates using RASSF1A and RASSF1C specific primers. All qRT-PCR reactions were set in triplicates and Cyclophillin was used as an internal loading control and used to normalize the relative expression levels using the 2^-ΔΔ ^method [42]. The RT-PCR analysis shows that RASSF1A expression was down and RASSF1C expression is up in established breast cancer cell lines compared the primary mammary epithelial cells.

### Identification of novel RASSF1C target genes

The observed increase in cell number in breast cancer cells over-expressing RASSF1C predicted that over-expression of RASSF1C might either down-regulate the expression of cell growth inhibiting/pro-apoptotic genes or up-regulate the expression of cell growth promoting/anti-apoptotic genes. Affymetrix-microarray analysis was performed using T47D cells over-expressing RASSF1C to answer this question. The control sample was RNA from T47D cells stably transduced with MLV-backbone (T47D-BB) and the experimental sample was RNA from T47D cells stably transduced with MLV-RASSF1C (T47D-1C). Prior to RNA isolation, T47D-BB and T47D-1C cells were treated with 1 μg/ml doxycycline for 48 hr. Data analysis was performed using the dChip program [21] and the thresholds for selecting significant genes were set at a relative difference > = 1.5-fold (or/and 2-fold), absolute signal difference > = 50, and p < 0.05. Genes that met all three criteria were considered as significant changes. Comparison results with False Discovery Rate (FDR) <5% was considered as a valid analysis.

**Figure 3:**
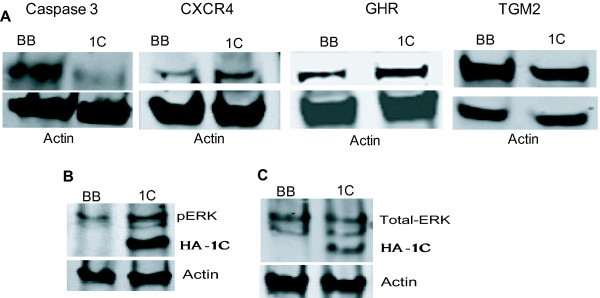
**(A) Western blot analysis of caspase 3, CXCR4, GHR, and TGM2 expression in T47D cells stably transduced with empty MLV-backbone (T47D-BB) or MLV-HA-RASSF1C (T47D-1C) using their corresponding antibodies**. The T47D cells were treated with 1 μg/ml doxycycline for 48 hr and used for Western blot analysis. It is clear that caspase 3 and TGM2 protein levels are down regulated and CXCR4 and GHR are up regulated in T47D cells over expressing RASSF1C validating the microarray and qRT-PCR findings (Table 1 and 2). Activation of ERK1/2 (B) and total ERK1/2 levels (C) were assessed in T47D-1C cells. The level of phosphorylated ERK1/2 in T47D-1C is much higher compared to that in T47D-BB while the total ERK1/2 protein levels are relatively the same in T47D-BB and T47D-1C.

We found that RASSF1C over-expression modulated the expression of a number of genes that are involved in cancer development, cell growth/proliferation, cell cycle, cell death, and apoptosis (Table 2). RASSF1C down-regulated several pro-apoptotic and tumor suppressor genes, including Bcl2-associated protein (BAX) [23], Caspase 3 [24], disabled homolog 2 (DAB2) [25], epithelial membrane protein 1(EMP-1) [26], insulin-like growth factor binding protein 3 (IGFBP-3) [27], mitochondrial tumor suppressor 1 (MTUSI) [28], ring finger protein 182 (RFN182) [29], SRY (sex determining region Y)-box 9 (SOX9) [30], sushi-repeat-containing protein, X-linked (SPRX) [31], transglutaminase 2 (TGM2) [32], and transmembrane protein 158 (TMEM158, also known as Ras-induced senescence 1(Ris-1)) [33]. RASSF1C also up-regulated several growth promoting genes that include apolipoprotein E (APOE) [34], carboxypeptidase E (CPE) [35], chemokine (C-X-C motif) receptor 4 (CXCR4) [36], human growth hormone receptor (GHR) [37], homeobox A1 (HOXA1) [38], muscle RAS (MRAS) oncogene homolog [39], SPANX family member A1 (SPANXA1), and SPANXB1 [40]. The RASSF1C target genes identified in this study are consistent with a potential growth promoting role for RASSF1C in breast cancer cells. We then selected several RASSF1C target genes and confirmed the microarray results using RT-PCR analysis (Table 3). We also show that changes in mRNA levels of caspase 3, CXCR4, GHR, and TGM2 are indeed translated to a change in protein expression in T47D cells (Figure 3A). We also found that T47D cell over-expressing RASSF1C displayed higher levels of phosphorylated ERK1/2 compared to control cells (Figure 3B). It should be noted that total ERK1/2 levels were the same in both T47D-BB and T47D-1C (Figure 3C). In addition, we show that silencing of endogenous RASSF1C expression in T47D cells resulted in an increase in caspase 3 and a decrease in CXCR4 mRNA expression (Table 4).

**Table 2:** Novel RASSF1C target genes in T47D breast cancer cells

Gene	Gene discription	Fold change
EFEM1	EGF-containing fibulin-like extracellular matrix protein 1	5.76
GHR	growth hormone receptor	4.3
EGR1	early growth response 1	5.9
TP53IN1	tumor protein p53 inducible nuclear protein 1	4.46
MRAS	muscle RAS oncogene homolog	3.8
MTUS1	mitochondrial tumor suppressor 1	-2
PROS	protein S (alpha)	4
EMP1	epithelial membrane protein 1	-7.5
HOXA1	homeobox A1	4.6
SEPP1	selenoprotein P, plasma, 1	7.92
MGMT	O-6-methylguanine-DNA methyltransferase	2.1
PAK1	p21/Cdc42/Rac1-activated kinase 1 (STE20 homolog, yeast)	2.1
CXCR4	chemokine (C-X-C motif) receptor 4	3.6
CREG	cellular repressor of E1A-stimulated genes 1	11.2
BMPR2	bone morphogenetic protein receptor, type II (serine/threonine kinase)	2
Noggin	Noggin	-3.6
SSP1	(osteopontin, bone sialoprotein I, early T-lymphocyte activation 1)	-14.5
TGM2	TGM2: transglutaminase 2 (protein-glutamine-gamma-glutamyltransferase)	3
SRPX	sushi-repeat-containing protein, X-linked	-7.25
SAMD5	sterile alpha motif domain containing 5	-11.47
MAP1B	microtubule-associated protein 1B	-11.7
RNF182	ring finger protein 182	-13.8
TMEM8	transmembrane protein 158	-11.29
LDB2	LIM domain binding 2	-5.85
IGFBP3	insulin-like growth factor binding protein 3	-4.96
COL131	collagen, type XIII, alpha 1	-8.69
FRMDB	FERM domain containing 4B	-5.2
SOX9	SRY (sex determining region Y)-box 9	-4.5
DAB2	disabled homolog 2, mitogen-responsive phosphoprotein (Drosophila)	-2.55
APOE	apolipoprotein E	3.74
CCND2	cyclin D2	-3
SPANX1	sperm protein associated with the nucleus, X-linked, family member A1	7.38
SPANX1	SPANX family, member B1	17.7
HLADP1	major histocompatibility complex, class II, DP alpha 1	12.7
HIST1H2BC	histone cluster 1 H2BC	5.34
DACH1	dachshund homolog 1 (Drosophila)	7.63
PCDH7	protocadherin 7	8.58
MYL9	myosin, light chain 9, regulatory	9
DPEP3	dipeptidase 3	10.66
CPE	carboxypeptidase E	11.47
LITAF	lipopolysaccharide-induced TNF facto	8.17
ABAT	4-aminobutyrate aminotransferase	10.82

List of novel RASSF1C target genes identified in T47D breast cancer cells using microarray analysis. Affymetrix-microarray analysis was performed using T47D cells over-expressing RASSF1C as outlined in Materials and Methods section. Data analysis was performed using the dChip program [21] and the thresholds for selecting significant genes were set at a relative difference > = 1.5-fold (or/and 2-fold), absolute signal difference > = 50, and p < 0.05. Genes that met all three criteria were considered as significant changes. Comparison results with False Discovery Rate (FDR) <5% was considered as a valid analysis.

**Table 3:** Validation of selected RASSF1C target genes

Gene	Fold Change
RASSF1C	252
Caspase 3	-3.3
CREG	4.9
CXCR4	5.4
EGR1	9.9
GHR	4.6
HOXA1	2.1
MRAS	4.8

List of selected RASSF1C target genes validated in the T47D breast cancer cell line using RT-PCR. The RT-PCR reactions were carried out in triplicates and the fold change was calculated using the 2^-ΔΔ^^C^_T _method [22]. Cyclophyllin was used as a loading control.

**Table 4:** Effects of silencing RASSF1C on target genes

Gene	Fold change
RASSF1C	-5
Caspase 3	+1.3
CXCR4	-2

Caspase3 and CXR4 gene expression in T47D cells treated with Mission^® ^lentiviral transduction particles specific to RASSF1C. The fold change is compared to T47D cells treated control scrambled shRNA (Mission^® ^lentiviral transduction control particles). The RT-PCR reactions were carried out in triplicate, and the fold change was calculated using the 2-ΔΔCT method [22]. Cyclophyllin was used as a loading control.

### RASSF1C over-expression enhances breast cancer cells invasion/migration in vitro

**Figure 4:**
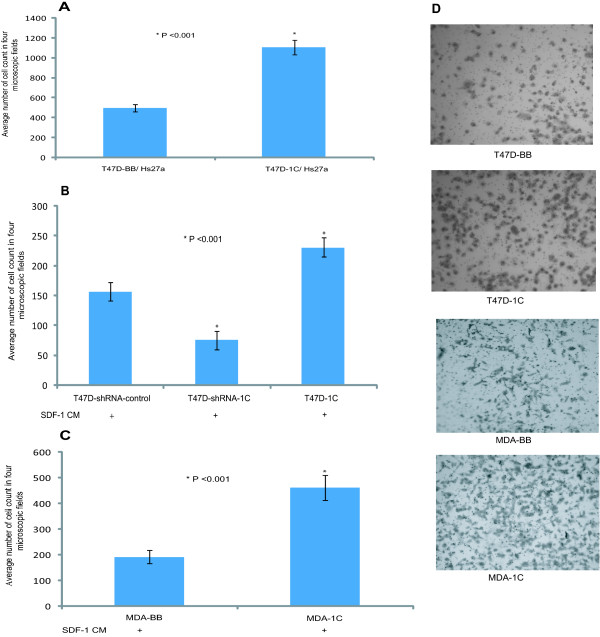
**(A) The BD BioCoatTM MatrigelTM Invasion Chamber was used to assess cell invasion/migration of T47D cells stably transduced with empty MLV-backbone (T47D-BB) or MLV-HA-RASSF1C (T47D-1C)**. T47D-1C or T47D-BB cells treated with doxycycline at 1ug/ml were co-incubated with Hs27a human stromal cells. Hs27a cells express the stromal derived factor-1 (SDF-1), the ligand for the CXCR4 receptor. After 24 hr incubation period the lower sides of the filters were fixed and stained and cells in four microscopic fields were counted. The average cell number count was plotted. T47D cells over-expression RASSF1C showed a higher number of cells invading the Matrigel chamber and migrating to the other side of the filter compared to T47D-cells, p <0.003. (B) T47D-lenti-shRNA control (Mission, Sigma), T47D-lenti-shRNA to silence RASSF1C (T47D-siRNA-1C) (Mission, Sigma), and T47D-1C cells were incubated with either non-conditioned serum-free medium or SDF-1-conditioned serum-free medium for 24 hr. Lower number T47D-siRNA-1C cells invaded the Matrigel chamber and migrating to the other side of the filter compared to T47D-siRNA control. (C) MDA-MB231-BB (MDA-BB) and MDA-MB231-1C (MDA-1C) cells were plated in the chamber inserts and placed in wells containing either non-conditioned serum-free medium or SDF-1-conditioned serum-free medium, and incubated for 24 hr. MDA-1C cells showed a higher number of cells invading the Matrigel chamber and migrating to the other side of the filter compared to MDA-BB cells, p < 0.001. The data suggests a potential role for RASSF1C in cancer cell metastasis, perhaps through the up-regulation of CXCR gene expression.

Because RASSF1C over-expression up-regulates the expression of CXCR4, a key metastasis gene, we carried out an *in vitro *invasion assay to determine if T47D cells over-expressing RASSF1C and grown in the presence of SDF-1 (the ligand for the CXCR4 receptor) were more invasive than control cells. For the invasion assays, T47D cells over-expressing RASSF1C were co-cultured with the human stromal cell line Hs27a, which secretes SDF-1, or were grown in conditioned serum-free medium containing SDF-I. Figure 4A shows that T47D-1C were significantly more invasive than the T47D-BB control cells. Interestingly, silencing of RASSF1C in T47D cells using lentiviral shRNA transduction particles significantly reduces T47D cell invasion/migration compared to cells infected with lentiviral shRNA control transduction particles (Figure 4B), further supporting that RASSF1C may promote breast cancer cell invasion/migration.

In addition to T47D cells, we also show that MDA-MB231 cells over-expressing RASSF1C were more invasive than the control cells (Figure 4C). All together, our novel findings suggest that RASSF1C may promote breast cancer cell invasion/migration perhaps in part through the up-regulation of the expression of the CXCR4 gene.

### RASSF1C over-expression attenuates apoptotic sensitivity in breast cancer cells

**Figure 5:**
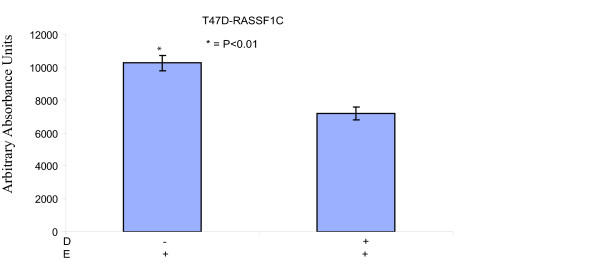
**Caspase 3 activity was measured in T47D cells stably transduced with MLV-HA-RASSF1C (T47D-RASSF1C)**. The T47D cells treated with doxycycline (D) and/or 45 um/ml etoposide (E) for 48 hr. Cells were subsequently assayed for caspase 3 activity. RASSF1C over-expression down regulates the expression of caspase 3 and hence less caspase 3 activity is observed in T47D-1C cells treated with both D and E compared to cells treated with E alone (p < 0.01). Data normalized to T47D cells that were treated with vehicle and the assay was carried at least three independent times with N = 4.

**Figure 6:**
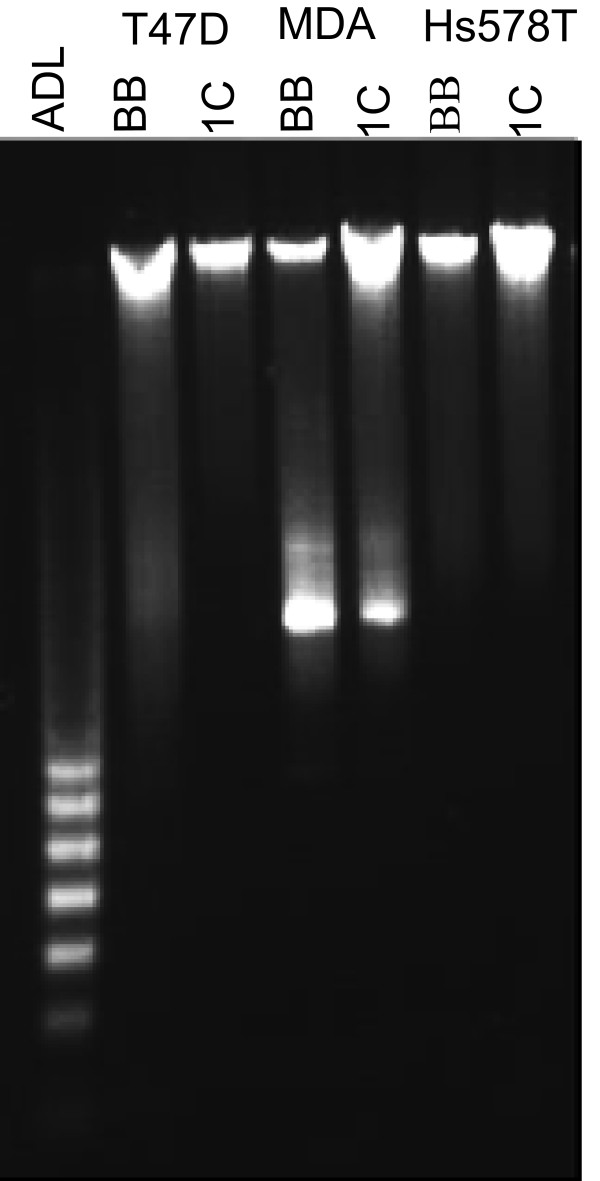
**T47D, MDA-MB231, and Hs578T cells stably transduced with an empty MLV-backbone (T47D-, MDA-, and Hs578T-BB) or MLV-HA-RASSF1C (T47D-, MDA-, and Hs578T-1C) vectors were treated with 1 μg/ml doxycycline for 14 days**. Genomic DNA was isolated for DNA fragmentation analysis using Apoptotic DNA ladder (ADL) kit. It is clear that DNA fragmentation did not occur in all the three cell lines tested suggesting that RASSF1C over-expression does not induce apoptosis.

Etoposide is a chemotherapy agent that is known to induce apoptosis through activation of caspase 3. Since over-expression of RASSF1C down-regulates caspase 3 expression, we hypothesized that over-expression of RASSF1C would reduce the amount of active caspase 3 that is induced by etoposide. We tested this hypothesis by measuring the amount of caspase 3 produced in response to etoposide by T47D breast cancer cells that either over-express or normally express RASSF1C. RASSF1C over-expressing cells (T47D-1C) exhibit reduced caspase 3 activity compared to cells that do not over-express RASSF1C (T47D-BB) when treated with etoposide (Figure 5). To further show that prolonged over-expression of RASSF1C does not promote apoptosis in breast cancer cells, DNA fragmentation analysis was carried out using genomic DNA isolated from breast cancer cells treated with 1 μg/ml doxycycline for 14 days. Over-expression of RASSF1C did not induce DNA fragmentation in breast cancer cells (Figure 6). These findings further support our hypothesis that RASSF1C plays a role in promoting breast cancer cell growth, and it may allow cancer cells to evade killing by chemotherapy agents.

## Discussion and Conclusions

The function of RASSF1C has not been as extensively studied as that of RASSF1A. Initial reports in the literature suggested that RASSF1C might function as a tumor suppressor in ovarian, prostate, renal cancer cells [15-17]. Recently, RASSF1C has been shown to interact with DAXX, a protein involved in apoptosis and transcriptional repression. It has been suggested that RASSF1C may contribute to the activation of Stress-Activated Protein-kinase/c-jun N-terminal kinase [17]. In contrast, we recently demonstrated that RASSF1C promotes lung cancer cell proliferation [19]. We previously showed that RASSF1C plays a role in promoting osteoblast cell proliferation through interaction with Insulin like Growth Factor Binding protein (IGFBP)-5 [18]. Consistent with our hypothesis, another group has recently shown that RASSF1C interacts with βTrCP (the receptor subunit of the SCF^βTrCP ^ubiquitin ligase that recruits signaling proteins and cell cycle regulators for proteosomal degradation) [41]. Through this mechanism RASSF1C over-expression in the human lung cancer cell line A549 promotes the accumulation β-catenin, an oncogene and a key player in the Wnt signaling pathway, leading to increased transcriptional activation and cell proliferation [41].

In this study, we demonstrated that reduction of RASSF1C mRNA in breast cancer cells correlated with a small but statistically significant decrease in cell proliferation compared to control cells that express RASSF1C. The reduction in RASSF1C did not affect cell viability as judged by trypan blue staining (data not shown). Overall our results are consistent with those we obtained in osteosarcoma (TE85, MG63, U2) and lung cancer (NCI H1299) cells [18,19]. In the studies with osteosarcoma cells, the effects of silencing RASSF1C in cells that express both RASSF1A and RASSF1C (TE85) were very similar to those observed in cells that express only RASSF1C (MG63 and U2), clearly indicating that RASSF1A does not modulate the effect of RASSF1C on cell proliferation and survival [18]. Based on these observations we think that, unlike RASSF1A, RASSF1C is not a tumor suppressor at least in breast, bone, and lung. Instead, it may function as a growth-stimulating factor.

To further determine the effect(s) of RASSF1C on breast cancer cell proliferation, we used a tetracycline-regulated MLV-based vector to stably over-express RASSF1C in MDA-MB231 and T47D. Tetracycline-regulated over-expression of RASSF1C did not inhibit cell proliferation. In fact, over-expression of RASSF1C caused reproducible and statistically significant increase in cell proliferation, further suggesting that RASSF1C is not a growth suppressor gene. In support of this, we have found that expression of RASSF1C is elevated in breast cancer cell lines compared to primary mammary epithelial cells, consistent with our hypothesis that RASSF1C may act as a potential growth and survival factor in breast cancer.

As mentioned above, a recent study suggested that regulated over-expression of RASSF1C may inhibit the growth of prostate cancer (LNCaP) and renal cell carcinoma (KRC/Y) cells [15]. However, we have now shown that RASSF1C over-expression does not inhibit cell growth but rather significantly increases proliferation of osteosarcoma [18], lung cancer [19], and breast cancer (this study) cells. The different findings related to the stimulatory function of RASSF1C in human osteosarcoma, breast cancer, and lung cancer cells [18,19,21,41], and the inhibitory function of RASSF1C in prostate, kidney, and ovarian cancer cells [15-17] may be due to tissue-specific actions of RASSF1C. It is well known that some proteins such as IGFBP-5 and FHIT (Fragile Histidine Triad) exert opposite effects in different tissues [42,43]. Our silencing and over-expression studies of RASSF1C underscores a potential growth promoting function at least in breast, lung, and osteosarcoma cell lines.

To begin to address the effect of RASSF1C on cellular growth, we carried out a microarray study to identify novel RASSF1C target genes. We found that RASSF1C over-expression interestingly modulated the expression of a number of genes that are involved in cancer development, cell growth/proliferation, cell cycle, cell death, and apoptosis. We validated the expression of several RASSF1C target genes by QRT-PCR and have also validated the expression of caspase 3, CXCR4, GHR, and TGM2 by Western blot analysis. We also found that cancer cells over-expressing RASSF1C exhibited increased phosphorylation of ERK1/2 compared to control cells (but no increase in total ERK1/2 levels), suggesting that RASSF1C may exert its activities in part through the activation of the ERK1/2 pathway. We also show that breast cancer cells over-expressing RASSF1C showed enhanced invasion/migration, while cells with knockdown of RASSF1C expression had reduced invasion/migration compared to control cells.

In addition, we found that RASSF1C over-expressing cells exhibited reduced caspase 3 activity compared to control cells when treated with etoposide. We also found that over-expressing RASSF1C for 14 days did not induce apoptosis as judged by lack of DNA fragmentation in Hs578T, MDA-MB231 and T47D cells, further suggesting that RASSF1C does not promote apoptosis. Taken together, the findings are consistent with our hypothesis that RASSF1C may potentially function as a metastasis promoting and apoptosis attenuating protein in breast cancer cells perhaps through the modulation of the CXCR4 and caspase 3 gene expression, respectively.

Our findings, together with others', are building a case that RASSF1C and RASSF1A have opposing, antagonistic functions at least in breast, lung, and bone cancer cells. For instance, it has been reported that RASSF1A inhibits lung cancer cell migration and promotes cell adhesion [44]. RASSF1A has also been shown to activate the pro-apoptotic Bax1 gene [45] which is consistent with its tumor suppressing activities. In this study, we have shown that RASSF1C promotes cell migration and down-regulates pro-apoptotic genes such as Bax, caspase 3, and SRPX. RASSF1C over-expression also up-regulates the expression of inhibitor of differentiation (Id2), which has been shown to be down-regulated by RASSF1A in nasopharyngeal carcinoma [46]. Also, RASSF1C down-regulates TGM2 gene expression, which is up-regulated by RASSF1A in the non-small cell lung cancer cell line A549 [47]. Lastly, our RASSF1C studies in breast cancer, lung cancer, and osteosarcoma cells, as well as other reports in the literature [41] also suggest that RASSF1C functions in an opposing manner to that of RASSF1A. In all of these studies, RASSF1C appears to stimulate cell growth, while RASSF1A is well established to suppress it. Thus, a fine balance of RASSF1A and RASSF1C isoform expression may be critical in determining the neoplastic potential of a cell.

**Figure 7:**
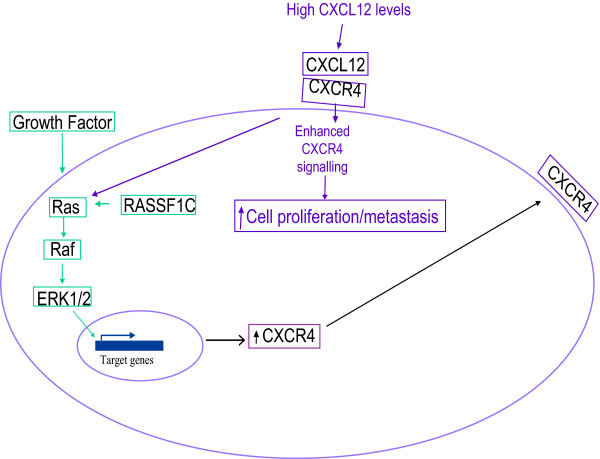
**A hypothetical model proposed to explain RASSF1C action in breast cancer cells: we hypothesize that RASSF1C activates the Ras-Raf-ERK1/2 pathway leading to up-regulation of the CXCR4**. Breast cancer cells expressing high number of CXCR4 receptor are attracted to migrate to secondary tissue sites where a cognate ligand (CXCL12) is expressed at higher levels (e.g., bone, liver, and lung).

As mentioned above microarray analyses of mRNA in cells subjected to forced expression of RASSF1C stimulates expression and silencing of RASSF1C reduces expression of genes that are associated with cell growth and proliferation. These data provide significant information to propose testable models to explain how RASSF1C exerts it growth promoting activities in breast cancer. For example, up-regulation of CXCR4 by RASSF1C is an interesting discovery suggesting the hypothesis that RASSF1C may play a role in promoting breast cancer metastasis as mentioned above. Recent studies have shown that CXCR4 expression is increased early in the transition from normal to transformed breast epithelium [48]. CXCR4 is a signaling receptor that interacts with a cognate ligand (SDF-1, also known as CXCL12) that is expressed at higher levels in tissues that attract breast cancer cells and support metastasis (e.g., bone, liver, lung). CXCR4 interaction with SDF-1 activates PI3K and Erk1/2 pathways and promotes cancer cell proliferation and metastasis [48,49]. In light of these findings we are proposing a testable model of RASSF1C action (see Figure 7) based on the hypothesis that RASSF1C increases the expression of CXCR4 in pre-malignant/malignant breast epithelial cells, thus supporting successful seeding and metastases in sites that express high levels of SDF-1. We plan to test this model and others to delineate the molecular mechanism/pathway(s) through which RASSF1C exerts its growth promoting effects on breast cancer cells using both *in vitro *and *in vivo *approaches.

In conclusion, together with our previous work on RASSF1C in osteosarcoma and lung cancer cells, our present findings in breast cancer cells provide further evidence that RASSF1C is not a tumor suppressor and instead it may promote metastasis and survival of cancer cells. Our studies further suggest that RASSF1C may accomplish this by up-regulation of specific growth promoting genes and down-regulation of specific pro-apoptotic genes.

## List of abbreviations

APOE: apolipoprotein E; ATM: ataxia telangiectasia mutated; BAX: Bcl2-associated protein; CPE: carboxypeptidase E; CXCR4: chemokine (C-X-C motif) receptor 4; DAB2: disabled homolog 2; Dox: doxycycline; EMP-1: epithelial membrane protein 1; ERK: extracellular receptor kinase; GHR: human growth hormone receptor; RA: Ras association; RASSF1: Ras association domain family 1; RT: reverse transcriptase; siRNA: small interfering RNA.

## Competing interests

The authors declare that they have no competing interests.

## Authors' contributions

MR participated in the design of the study, contributed to data analysis, and drafting of the manuscript. SB carried tissue culture and gene cloning work. MB carried out the western blot analysis and apoptosis assays. SC prepared and viral vectors and viral cell transduction. JM performed cell tissue culture work and cell invasion assays. RA performed RNA and RT-PCR work. XL carried out microarray hybridization and analysis. DS participated in RT-PCR design and contributed to data analysis. SM participated in drafting the manuscript. YA designed and supervised the study, carried out the gene silencing and over-expression work, and contributed to data analysis and drafting of the manuscript. All authors read and approved the final manuscript.

## Pre-publication history

The pre-publication history for this paper can be accessed here:

http://www.biomedcentral.com/1471-2407/10/562/prepub
